# Evaluating Osteoarthritis Severity in Mice Using μCT-Derived Geometric Indices

**DOI:** 10.3390/biology15030262

**Published:** 2026-01-31

**Authors:** Churou Tang, Chutamath Sittplangkoon, Cheng Xiang, Lindsay Schnur, Rong Duan, Xi Lin, Dongmei Li, Zhenqiang Yao

**Affiliations:** 1 Department of Pathology & Laboratory Medicine, University of Rochester Medical Center, 601 Elmwood Avenue, Rochester, NY 14642, USA; 2 Center for Musculoskeletal Research, University of Rochester Medical Center, 601 Elmwood Avenue, Rochester, NY 14642, USA; 3 Clinical and Translational Science Institute, University of Rochester Medical Center, 265 Crittenden Boulevard CU 420708, Rochester, NY 14642, USA

**Keywords:** osteoarthritis, micro-computed tomography, structural parameter, geometric indices, quantitative index

## Abstract

Imaging is the gold standard for diagnosing osteoarthritis (OA). However, it remains challenging to precisely assess OA in murine models. The aim of this study is to establish μCT-based geometric indices for assessing the disease severity of post-traumatic OA (PTOA) and age-related OA (AROA). Following medial meniscectomy (MMS) in adult C57BL/6 mice, distal femoral length remained unchanged, whereas its width increased significantly. As a result, distal femoral width-to-length ratio was markedly elevated at 4 and 8 weeks after MMS (1.33 ± 0.05 and 1.47 ± 0.1, n = 7 and 5 joints, respectively) compared to contralateral normal joints (1.19 ± 0.04, n = 9), yielding an area under the curve (AUC) of 1.0 (95% CI, 1.0–1.0). Similarly, the tibial secondary ossification center (IIOC) height decreased, whereas its width increased after MMS. Consequently, the tibial IIOC height-to-width ratio was significantly reduced at 4 and 8 weeks post-MMS (0.25 ± 0.02 and 0.24 ± 0.02, respectively) compared with a normal joint (0.304 ± 0.011), with an AUC of 1.0 (95% CI, 1.0–1.0). Bland–Altman analysis demonstrated strong interrater reproducibility, with intraclass correlation coefficients of 0.853 (95% CI, 0.681–0.936) for the femoral ratio and 0.887 (95% CI, 0.748–0.952) for the tibial ratio. Notably, these MMS-induced changes in femoral and tibial geometric indices were attenuated in mice with genetic deletion of GM-CSF, a key mediator of OA. Importantly, μCT-derived geometric indices were validated by histological analysis. Furthermore, increased distal femoral width-to-length ratio and reduced tibial IIOC height-to-width ratio were also observed in the knee joints of 28-month-old mice with AROA. In addition, an enlarged patella and a calcified synovium–capsule serve as a reference for AROA and PTOA. Collectively, μCT-based geometric indices are useful and easily quantifiable metrics for assessing disease severity and therapeutic response of PTOA and AROA models in murine. Of note is that the distal femoral and proximal tibial geometric indices were developed based on severe OA induced by MMS, and their applicability to mild OA requires further investigation.

## Simple Summary

Murine models of osteoarthritis (OA) are essential for investigating disease mechanisms and for the preclinical development of disease-modifying therapies. However, reliable, easily quantifiable, and objective metrics for assessing OA disease severity in murine models are lacking. This study addresses this gap by developing μCT-derived geometric indices in mice with knee-joint OA. We found that the distal femoral width-to-length ratio increased significantly at 4 and 8 weeks following medial meniscectomy (MMS) in 10–12-month-old middle-aged mice, reflecting osteophyte formation at the distal femur. In contrast, the proximal tibial height-to-width ratio decreased significantly, reflecting articular cartilage degeneration, subchondral bone collapse, and osteophyte formation at the proximal tibia. Similar changes in these geometric indices were also observed in 28-month-old aged mice with knee OA. Importantly, these indices demonstrated strong inter-rater reproducibility, and MMS-induced changes were attenuated in mice with genetic deletion of GM-CSF, a key mediator of OA. Notably, both indices remained unchanged in normal knee joints from young-to-middle-aged mice, enabling robust comparisons across experiments. Collectively, these μCT-based geometric indices provide reproducible and easily quantifiable metrics that overcome challenges in directly quantifying osteophytes and reconcile contradictory μCT-based subchondral bone mass measurements, thereby improving the precision of OA diagnosis and disease severity assessment.

## 1. Introduction

Osteoarthritis (OA) is the most common joint disease, affecting millions of people in the world [1,2]. It is also common for companion animals such as dogs [3] and cats [4]. The hallmarks of OA are the irreversible loss of articular cartilage and excessive joint ossification, including osteophyte formation and subchondral sclerosis [5,6]. Joint motion in OA is therefore limited or lost and is associated with severe pain. One of the major goals in orthopedic research is to develop a disease-modifying therapy to reverse or stop the pathological process of OA. Murine OA models are essential for investigating disease mechanisms and for the preclinical development of disease-modifying therapies. However, there is currently no reliable objective tool that can accurately and sensitively quantify cartilage loss and excessive ossification to evaluate the effectiveness of a novel therapeutic approach in murine OA models.

Radiography remains the golden standard and is essential to diagnose OA clinically [7]. The key radiographic features of human OA include osteophyte formation on the margins or centrals of the joint, subchondral cysts (in early stage), or sclerosis, asymmetric joint space narrowing between the bones of the joints that is depicted reflecting cartilage loss and meniscal damage and extrusion [8,9]. However, plain radiography is not sensitive to assess OA severity. It cannot directly visualize the articular cartilage and is difficult to ascertain the optimum and reproducible position of the joint in serial assessments. In addition, X-rays cannot directly detect synovial inflammation and hyperplasia.

Magnetic resonance imaging (MRI) is a powerful non-invasive diagnostic imaging technique to detect cartilage degeneration, bone marrow and meniscal lesions, and synovitis [10,11]. Thus, an MRI can monitor the effects of conservative and surgical therapy in humans [12], enabling study of the potential OA risk factors and mechanisms of the OA disease process [13]. Murine MRI generally achieves spatial resolutions in the range of approximately 100–150 µm voxel dimensions [14,15]. However, this resolution is insufficient for accurately quantifying the fine structural details of mouse joints. The key inherent limitation of MRIs is that they cannot define the cortical and trabecular bone and osteophyte.

Computed tomography (CT) is excellent for showing bone, osteophytes, and osteolytic cysts [16]. For decades, micro-CT (μCT) has been the gold standard for analyzing bone microarchitecture and mineral density in small animals [17]. It is also used to evaluate the disease severity of OA in mice. The most commonly used parameter is the subchondral bone mineral density [18,19,20], which reflects subchondral bone osteosclerosis in late-stage OA. However, subchondral bone loss is also commonly associated with OA [21,22]. Subchondral sclerosis and osteopenia represent two opposing biological processes. Therefore, neither alone is sufficient to assess OA severity. The quantification of osteophyte volume can be conducted [23], but it is challenging to define the boundary between the osteophyte and normal bone, and the ossa sesamoidea around the joints are commonly identified as separated osteophytes. Quantification of the whole epiphyseal volume has also been used to assess the osteophyte [23]. However, this approach faces challenges similar to those of direct osteophyte measurement, as there is no well-defined anatomical landmark to delineate the entire epiphysis. Therefore, quantified data on osteophyte volume is less frequently reported in mouse models of OA.

Taken together, there are currently no reproducible, sensitive, and easily quantifiable metrics for assessing disease severity in murine OA models. The aim of this study is to evaluate whether μCT-derived geometric indices can address this gap in mice with knee-joint OA. Our findings suggest that the geometric indices of the distal femur and proximal tibia serve as reliable, reproducible, and sensitive parameters for diagnosing and evaluating OA severity and therapeutic efficacy. In particular, the normalized measures, distal femoral width-to-length ratio and proximal tibial height-to-width ratio at the tibial secondary ossification center (IIOC), defined as the region from articular surface to growth plate, enable robust comparisons across experiments and address an important methodological gap in the OA research.

## 2. Materials and Methods

### 2.1. Animals and Study Design

In-house-bred C57BL/6 and GM-CSF knockout (JAX strain #026812) mice, aged 10 to 12 months, chosen randomly by a technician who did not conduct the experiments, from the pool of mice with similar ages, including both genders, underwent medial meniscectomy (MMS) surgery on the right knee, while the left knee served as the normal control. For the 8- and 4-week time points following MMS, the experiments were conducted independently, whereas the normal control joints were combined. The numbers of mice were 7 and 6 for WT and 5 and 6 for GM^−/−^ at 8 and 4 weeks post-MMS, respectively. For the combined controls, only three normal joints from each genotype were randomly selected for μCT scanning and histological examination at the time point of 8 weeks post-MMS. Statistical analysis confirmed that none of the geometric indices differed among the sets of normal joints. Aged (28 months) and young adult (5 months) C57BL/6 background mice, obtained from the National Institute on Aging (NIA), were used to validate whether the geometric parameters derived from μCT imaging are suitable for evaluating the severity of age-related OA (AROA). The 28- and 5-month mice are equivalent to humans aged late 70s–early 80s and mid-20s, respectively [24]. The skeletal system in the 5-month mice is fully mature. Both right and left knee joints from four young or aged mice, randomly selected from the same ordered batch, 8 joints per group, were included in the study. All animal experimental protocols were approved by the University of Rochester Committee for Animal Resources (protocol number: UCAR-2011-043E), and all methods were carried out in accordance with the guidelines and regulations of the American Veterinary Medical Association (AVMA). Mice were housed in a specific pathogen–free animal facility under controlled environmental conditions, including a 12 h light/12 h dark cycle, ambient temperature of 20–24 °C, and relative humidity of 40–60%. Animals had ad libitum access to standard laboratory chow and water. Buprenorphine SR was administered subcutaneously at a dose of 1.0 mg/kg 5 min prior to MMS surgery, which provides continuous analgesia for up to 72 h.

### 2.2. Micro-Computed Tomography (μCT) Imaging and Analysis

Both right and left legs were fixed in 10% neutral buffered formalin for 48 h and were transferred to 70% ethanol at 4 °C for storage. The knee joints were scanned using a Scanco Medical VivaCT 40 cone-beam CT (Scanco Medical, Bassersdorf, Switzerland) at 10.5-micron isotropic (cubical) voxels, 55 kVp, 145 µA, and 300 msec integration time. The machine was set at a threshold of 220, as used in our center and other institutions [25,26] to distinguish bone from soft tissues. This threshold was initially determined visually by a trained technician and then adjusted until the bony microarchitecture of the ROI closely matched that observed in the unthresholded X-ray image. Scanco evaluation software V6.5 was used to conduct the analysis based on the drawn contours of regions of interest (ROI), following the standard of operation at the Center of Musculoskeletal Research (CMSR) by a scientist who was not informed of any experimental information or group allocation.

General three-dimensional images were obtained with simple segmentation at a Scanco threshold of 320, which was determined on inspection of the first specimen. This translates to a linear attenuation coefficient of 2.56 cm^−1^.

A Scanco threshold of 270 (2.16 cm^−1^) is used to define the tibial subchondral plate and cortical bone. The region from the tibia articular surface to the tibia growth plate is defined as the beginning at the most proximal appearance of the articular surface, proceeding distally until the most proximal appearance of the growth plate. All bone in the region is included.

Tibia subchondral analysis begins by starting at the knee and proceeding distally until the cortical shell is penetrated. All non-cortical bone is included, both solid and trabecular. Proceeding distally, all bone is included in the growth plate. The growth plate is excluded. At that point, the solid bone transitions to cortical bone and is excluded. The trabecular bone is included until the end of the growth plate is reached, which begins the trabecular region. Medial and lateral regions were determined visually through the center of the proximal tibia. A Scanco threshold of 270 (2.16 cm^−1^) is used. Cancellous bone volume (BV/TV, %), trabecular thickness (Tb.Th, µm), trabecular number (Tb.N, #/mm), and trabecular separation (Tb.Sp, µm) are calculated according to standard guidelines [17].

The tibial IIOC height, defined as the distance between the growth plate and the articular surface, was calculated by counting the μCT slices between the most proximal appearance of the articular surface and the most proximal appearance of the growth plate. Similarly, tibial subchondral plate thickness [23] was calculated by counting μCT slices from the articular surface to the first presence of subchondral trabecular bone. During μCT scanning, the legs were positioned parallel to the long axis of tibias within the tubes to minimize the influence of tibial tilt on the measurements, although the image orientation was not further corrected. Each slice is 10.5 µm. The reported μCT dataset is included in Supplemental Table S1.

### 2.3. Analysis of Geometric Parameters in Distal Femoral and Proximal Tibia by Amira Software

To analyze the geometric parameters in murine knee joints, μCT datasets (dcm file format) were imported into Amira software (v.2020.2, ThermoFisher Scientific, Hillsboro, OR, USA). A marker-based watershed segmentation method was utilized to construct 3D models of the knee joint. A 3D median filter was first applied to the original dataset to denoise the image. Following murine hind-paw μCT bone segmentation methods developed by Kenney et al. [27], a set threshold (>2500 Hounsfield Units (HU)) was applied through Interactive Thresholding to the dataset to define bone relative to the surrounding air and soft tissue, generating a binary mask of the bones [27,28,29]. This threshold was not scanner-dependent. On the median filtered dataset, further segmentation was achieved by the placement of watershed markers, which are pixel regions with a relatively constant signal intensity identified as unique objects. To create watershed markers, semi-automated segmentation (Magic Wand tool) was performed. A seed voxel was first manually chosen based on the general region of each bone in a midsagittal ortho section. The Magic Wand tool with all-slices was then used to perform a 3D region-growing operation to automatically select the largest connected area that contains the seed voxel and all voxels with gray values within the empirically found best threshold (3000 HU to 5000 HU).

Separate objects were labeled as individual materials, functioning as markers. Using the Marker-Based Watershed Inside Mask algorithms, a bone-specific segmentation that expands to the full volume of the bone was created. The Extract Label module was then applied to the result of the Marker-Based Watershed Inside Mask [18,27], isolating the desired bone for analysis of the knee joint, including the femur, tibia, patella, and menisci. The Volume-Rendering module was used to visualize the dataset, generating a 3D model for measuring geometric parameters. The Material Statistics module was applied to the watershed segmented results to retrieve the volume of the patella and menisci.

Measurements of femoral geometric parameters were performed using the interactive distance measurement (Ruler tool) in Amira on the constructed 3D model. The length of the distal femur was measured as the straight-line distance between the middle point of the intercondylar groove upper line and the intercondylar notch. The intercondylar groove is the femoral surface of the patellofemoral joint. The intercondylar notch, located between the femoral condyles, serves as the attachment site for the anterior cruciate ligament (ACL) and posterior cruciate ligament (PCL) within the knee joint. When the intercondylar notch was deeply eroded, the measurement point was defined at the edge of the notch entrance, where it intersected with the surface of the surrounding healthy bone at the distal femur. The width of the distal femur was measured as the distance between the edges of the lateral and medial condyle, representing osteophyte formation. Manual definition of the two voxels for each measurement allows for retrieval of the straight-line distance between the two voxels under the Measurement module.

Tibial width and height in IIOC at the level of the growth plate were measured on ortho slices after image orientation was corrected. When the μCT images were rotated, the original μCT dataset was transformed using the Transform Editor to the global center to create a new dataset oriented parallel to the x or y plane. The *Ortho Slice* module was applied to the newly transformed dataset, resulting in a frontal section slice of the tibia. The correction was listed in Supplemental Table S2. The ortho slice with the maximum height of tibial subchondral compartments was used for measurement after viewing all scanned slices. The interactive distance measurement (Ruler tool) was utilized to measure the tibial geometric parameters: the width of the tibia and height of the medial and lateral subchondral compartments of the tibia. The width of the proximal tibia was defined as the straight-line distance between the borders of the medial and lateral tibial condyles at the level of the growth plate. This measurement was obtained from the slice in which both the medial and lateral femoral condyles were clearly visualized, and the distance between them was maximal after review of all scanned slices. The dataset of geometric indices from the distal femur and proximal tibia is listed in Supplemental Table S3. The 3D image construction, orientation correction, and the geometric index measurements were conducted independently by two investigators who did not know the animal group allocation, surgery, or any treatment.

### 2.4. Histological Analysis of the OA

After μCT scanning, the legs were decalcified for 3 weeks using 10% EDTA at 4 °C, then processed and embedded in paraffin in the sagittal plane, with the medial side of the joints facing the surface. The paraffin blocks were trimmed to obtain wide exposure of both the tibial and femoral condyles and observed under a microscope. When the femoral and tibial articular surfaces were in close proximity, 4 µm-thick tissue sections were collected and serially cut, with two sections mounted on each microscope slide, for a total of 24 slides. Eight slides were considered as one level. The first slide of each level was stained with Alcin blue, followed by H&E (ABH) or with H&E alone. All stained slides, including both diseased and normal joints, were numbered to allow blinded evaluation of the histomorphometry parameters by an experienced observer who was not involved in group allocation, surgical procedure, and aging, performed twice, as we reported [30].

### 2.5. Statistical Analysis

All results are given as the mean ± S.D. All data were tested for normality using the Shapiro–Wilk test in GraphPad Prism (Version 6.07). Comparisons between two groups were analyzed using a two-tailed unpaired Student’s *t* test, and comparisons among three or more groups were analyzed using one-way analysis of variance (ANOVA) followed by Dunnett’s post hoc multiple-comparisons test when data were normally distributed. When the data were skewed, log-transformed data were used for statistical analysis. All analyses were performed using a two-tailed significance level of 0.05. The *p* values < 0.05 were considered statistically significant. GraphPad Prism was used to perform ROC analysis to evaluate the performance of geometric indices in diagnosing OA. Bland–Altman plot and intraclass correlation coefficient were used to assess inter-rater reproducibility. The Benjamini–Hochberg (BH) procedure was used to control the false discovery rate (FDR) for multiple comparisons within the generalized linear model framework using statistical analysis software R 4.5.2 (R Core Team, 2025, R Foundation for Statistical Computing, Vienna, Austria). Power analysis was performed based on one-way ANOVA. Based on our preliminary data, the mean maximal heights of the tibial IIOC in WT control, WT MMS at 4 weeks, GM^−/−^ control, and GM^−/−^ MMS at 4 weeks groups were 0.849, 0.756, 0.895, and 0.9387 mm, respectively, with corresponding standard deviations of 0.035, 0.063, 0.085, and 0.082 mm. A sample size of five mice per group was required to achieve 85% power at a two-tailed significance level of 0.05.

## 3. Results

### 3.1. The μCT Imaging Detection of Tibial Subchondral Bone Alternation Following MMS

**Figure 1:**
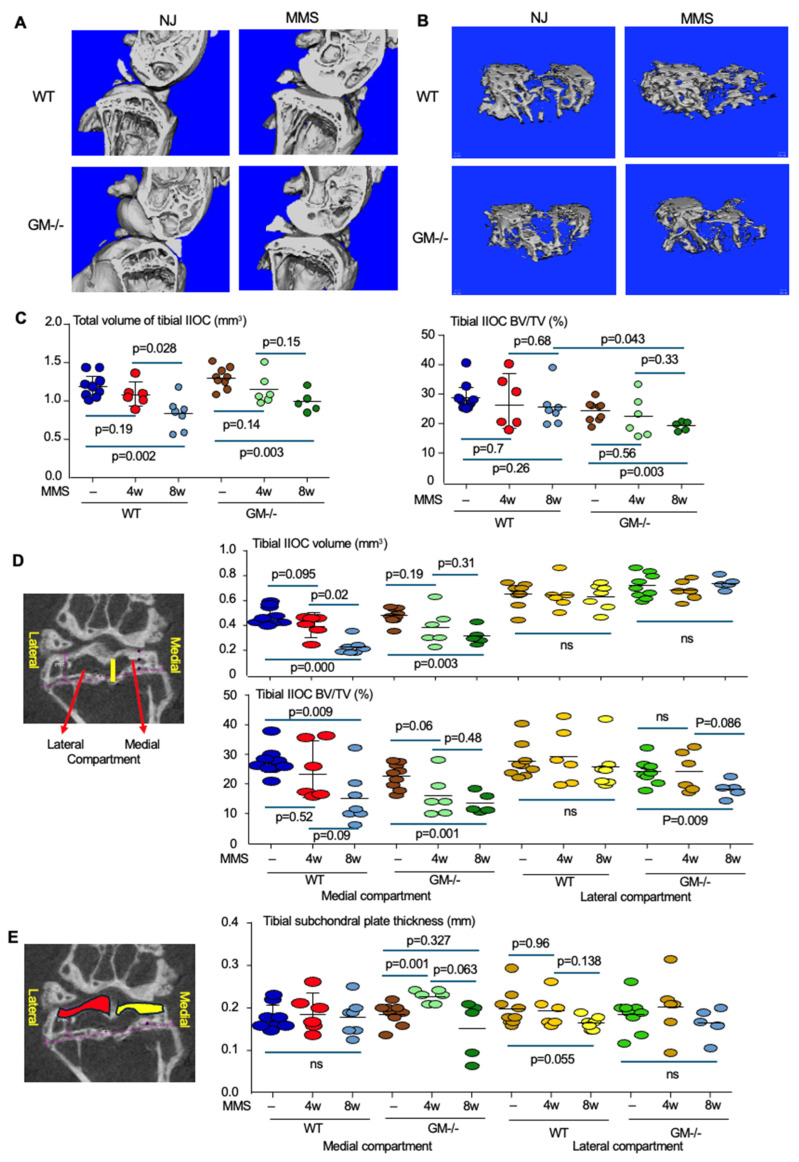
The μCT analysis of tibial subchondral bone following MMS in the knee joints of WT and GM^−/−^ mice. (**A**) Representative μCT images of coronal plane of the knee joints after 8 weeks of MMS. (**B**) Representative coronal plane of μCT image showing the subchondral bone in the tibial IIOC after 8 weeks of MMS. (**C**) Decreased total volume of tibial IIOC for both WT and GM^−/−^ mice at 4, but not 8, weeks post-MMS. Unchanged bone volume fraction (BV/TV) in WT mice following MMS, while GM^−/−^ mice have reduced BV/TV in IIOC after 8, but not 4, weeks of MMS. (**D**) Medial and lateral compartments of tibial IIOC were determined visually through the curved subchondral and growth plate (yellow line in the image) to calculate the volume and BV/TV in the medial and lateral compartments of tibial IIOC after 4 and 8 weeks of MMS. (**E**) The subchondral plate thickness (the distance between the tibia articular surface and the first presence of trabecular bone) was calculated by counting the μCT slice number in the medial (yellow area) and lateral (red area) compartments of tibial IIOC after 4 and 8 weeks of MMS. One-way ANOVA+/Dunnett test was used for the statistical analysis. Sample number: normal joints (NJ), n = 9 per group for both WT and GM^−/−^ mice (3 males and 6 females in each group); 4 weeks post-MMS, n = 6 per group for both WT and GM^−/−^ mice (3 each gender); and 8 weeks post-MMS, n = 7 WT female mice and n = 5 GM^−/−^ female mice.

We found that the bone volume fraction (BV/TV) in the tibial subchondral space of the right knee joints subjected to medial meniscectomy (MMS) showed no significant change at 4 or 8 weeks compared to the contralateral normal knee joints in the WT mice (Figure 1A–C). In contrast, the total tibial subchondral volume in the knee joints with MMS was significantly reduced after 8 weeks and showed no difference after 4 weeks, relative to that of contralateral normal joints in the WT mice (Figure 1A–C). Similarly, the total tibial subchondral volume in the knee joints in GM^−/−^ was reduced at 8, but not 4, weeks after MMS compared to the contralateral normal joints (Figure 1A–C). In addition, the BV/TV in the tibial subchondral space was reduced at 8, but not 4, weeks after MMS (Figure 1A–C). Further analysis indicates that the reduction in total tibial subchondral bone volume following MMS is primarily due to a decrease in the medial, but not the lateral, subchondral compartment in both WT and GM^−/−^ mice (Figure 1D), consistent with medial meniscectomy. In addition, the BV/TV in the medial subchondral compartment was significantly reduced in both WT and GM^−/−^ mice at 8 weeks post-MMS compared to their respective normal joint control (Figure 1D).

The thickness of the medial subchondral bone plate, a thin and dense layer of bone located just beneath the articular cartilage [31,32], in the tibia of WT mice showed no significant changes at either the early (4 weeks) or late (8 weeks) stages of OA induced by MMS (Figure 1E). In contrast, the lateral subchondral bone plate thickness of the tibia decreased marginally in WT mice at 8 weeks post-MMS compared to normal joints (Figure 1E). For GM^−/−^ mice, medial subchondral bone plate thickness increased temporarily at 4 weeks and then decreased marginally at 8 weeks post-MMS, while the lateral subchondral bone plate thickness remained unchanged.

### 3.2. The μCT Imaging Quantification of the Geometric Parameters in Distal Femur

**Figure 2:**
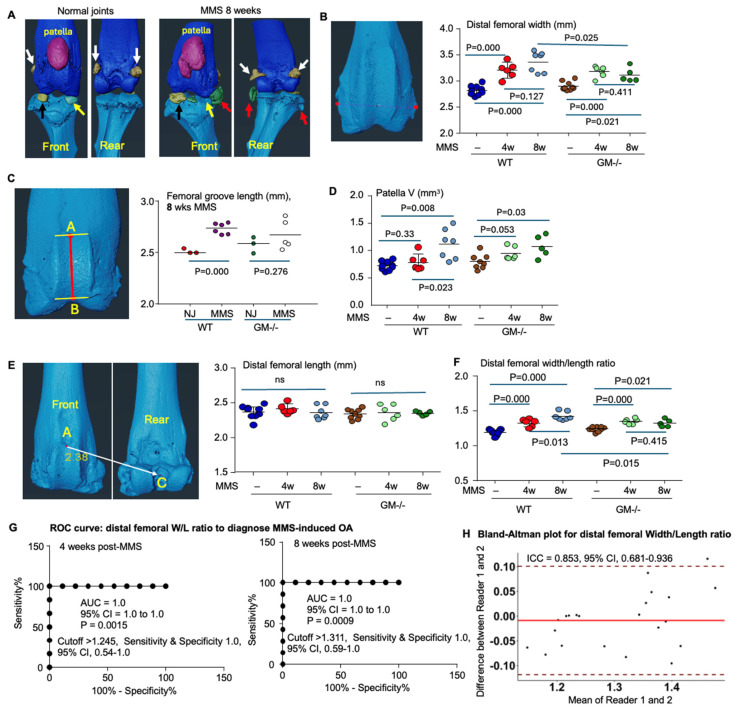
Geometric parameters of the distal femur after MMS in WT and GM^−/−^ mice. Three-dimensional reconstructions of µCT-scanned knee joints were generated using Amira software. (**A**) Segmentation of the patella (purple), ossa sesamoidea (white arrows), lateral meniscus (black arrows), medial meniscus (yellow arrows), and osteophytes (red arrows) in front and rear views of µCT images from OA joints at 8 weeks post-MMS, compared with a normal control joint (NJ). (**B**) Measurement of distal femoral width (distance between the lateral and medial condylar edges) and the quantitative data in normal joints and OA joints from WT and GM^−/−^ mice at 4 weeks after MMS. (**C**) Femoral groove length, defined as the distance between the upper and lower midpoints of the femoral groove line, as shown the red line from point A to B, in normal joints versus OA joints at 8 weeks post-MMS. (**D**) Patellar volume at 4 and 8 weeks after MMS, quantified from the segmented images shown in panel (**A**). (**E**) Schematic illustration of distal femoral length measurement, defined as the distance from the midpoint of the upper femoral groove line to the intercondylar notch, shown as the white arrow from point A to C. (**F**) Distal femoral width-to-length ratio in normal joints and OA joints at 4 and 8 weeks post-MMS. (**G**) Receiver operating characteristic (ROC) analysis showing the area under the curve (AUC) and cutoff value of distal femoral width-to-length ratio in diagnosing OA at 4 and 8 weeks post-MMS in WT mice. (**H**) Bland–Altman plot and intraclass correlation coefficient (ICC) for distal femoral width-to-length ratio of the data of WT mice with MMS and normal control. Statistical analysis was performed using one-way ANOVA with Dunnett’s post hoc test. Sample number: NJ n = 9 per group for both WT and GM^−/−^ mice (3 males and 6 females in each group); 4 weeks post-MMS, n = 6 per group for both WT and GM^−/−^ mice (3 each gender); and 8 weeks post-MMS, n = 7 WT female mice and n = 5 GM^−/−^ female mice.

From the front view of the micro-CT images, the transverse width of the distal femora appeared to be markedly increased (Figure 2A). Thus, the distal femora were segmented using Amira software to measure distal femoral width, the distance between the lateral and medial condyle edges, representing osteophyte formation, on the front view of μCT images. Indeed, the distal femoral width was significantly increased in both WT and GM^−/−^ mice at 4 and 8 weeks after MMS compared to their respective contralateral normal control joints (Figure 2B). Notably, distal femoral width in normal knee joints did not differ between the GM^−/−^ and WT mice. Distal femoral width increased less in GM^−/−^ mice, resulting in greater distal femoral width in GM^−/−^ mice than in WT mice at 8 weeks post-MMS (*p* = 0.025, Figure 2B).

Adult bone size varies significantly, even within the same litter. Furthermore, continuous bone remodeling, leading to increased diameter post-growth, complicates comparisons across experiments. To address this, we aim to normalize distal femoral width using a constant longitudinal parameter, enabling robust comparisons across experiments.

One longitudinal parameter is the length of the femoral groove, forming the patellofemoral joint. The femoral groove length, defined as the distance between the midpoints of the upper and lower femoral groove lines, was significantly increased at 8 weeks post-MMS compared with the contralateral normal knee joints in the WT mice (Figure 2C). Consistent with this, the patella volume was significantly enlarged in the right knee with MMS compared to that in normal joints at 8 but not 4 weeks post-surgery in the WT mice (Figure 2D). The patella volume also increased in GM^−/−^ mice marginally at 4 weeks and significantly at 8 weeks following MMS compared to their normal control joints (Figure 2D).

The second longitudinal parameter, distal femoral length, is defined as the distance between the midpoint of the upper femoral groove line and the intercondylar notch of the femur (Figure 2E). We found that distal femoral length remained unchanged in either WT or GM^−/−^ mice compared to their respective contralateral normal control joints at 4 or 8 weeks post-MMS (Figure 2E), indicating that it can serve as a stable reference parameter to normalize other geometric measurements for comparing OA severity across experiments. Interestingly, in WT mice, the distal femoral width-to-length ratio significantly increased at 4 weeks post-MMS (1.33 ± 0.05, *p* < 0.001) and showed a further pronounced increase by 8 weeks (1.47 ± 0.1, *p* < 0.001) compared to their contralateral normal joints (1.19 ± 0.04) (Figure 2F). The distal femoral width-to-length ratio remained below 1.24 in all nine normal knee joints from WT mice, whereas this value was over 1.3 in all joints with MMS following 4 and 8 weeks (Figure 2F).

A receiver operating characteristic (ROC) analysis demonstrated an area under the curve (AUC) of 1.0 for both 4 and 8 weeks post-MMS (95% confidence interval [CI], 1.0–1.0, *p* < 0.01) (Figure 2G). This perfect discriminative ability was achieved with distal femoral width-to-length ratio cutoff values of >1.245 at 4 weeks and >1.312 at 8 weeks post-MMS, each yielding 100% for both sensitivity and specificity (Figure 2G). A second rater independently measured the geometric parameters of the distal femur. Importantly, the distal femoral width-to-length ratio demonstrated a strong interrater reproducibility in diagnosing MMS-induced OA, with an intraclass correlation coefficient (ICC) of 0.853 (95% CI, 0.681–0.936) shown by Bland–Altman analysis (Figure 2H).

The distal femoral width-to-length ratio in normal control knees did not differ between the GM^−/−^ and WT mice. In the GM^−/−^ mice, this ratio increased significantly at 4, but not 8, weeks following MMS (Figure 2F). Consequently, at 8 weeks post-MMS, GM^−/−^ mice exhibited a marginally lower distal femoral width-to-length ratio compared to WT mice (Figure 2F).

### 3.3. Quantification of Geometric Parameters in Tibial Secondary Ossification Center (IIOC) Following MMS by μCT Imaging

**Figure 3:**
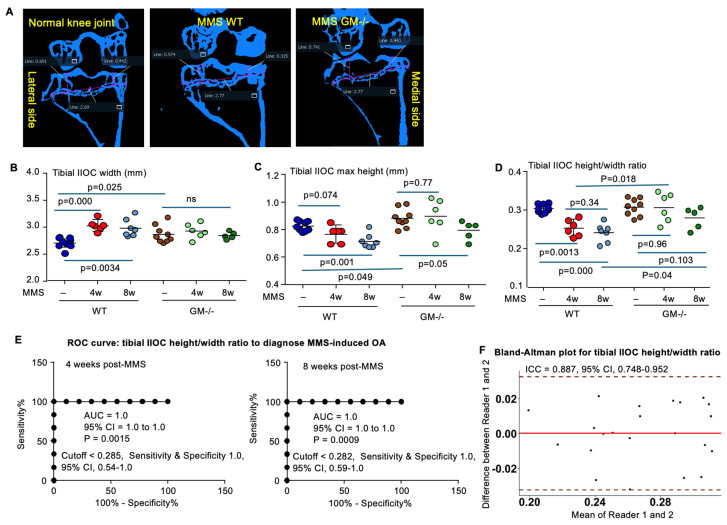
Geometric parameters of the proximal tibia after MMS in WT and GM^−/−^ mice. (**A**) µCT images of the coronal plane of knee joints at 8 weeks after MMS, after alignment correction, showing the measurement of tibial IIOC width at the level of the growth plate and the heights of the medial and lateral compartments of tibial IIOC. (**B**) Changes in tibial IIOC width at 4 and 8 weeks post-MMS. (**C**) Maximal height of tibial IIOC, determined by counting the number of µCT slices between the most proximal appearance of the articular surface and the most proximal appearance of the growth plate (10.5 µm per slice). (**D**) Change in tibial IIOC height-to-width ratio following MMS in WT and GM^−/−^ mice. (**E**) ROC analysis showing the AUC and cutoff value of tibial IIOC height-to-width ratio in diagnosing OA at 4 and 8 weeks post-MMS in WT mice. (**F**) Bland–Altman plot and ICC for tibial IIOC height-to-width ratio from the data of WT mice with MMS and normal control. Statistical analysis was performed using ANOVA with Dunnett’s post hoc test. Sample number: NJ n = 9 per group for both WT and GM^−/−^ mice (3 males and 6 females in each group); 4 weeks post-MMS, n = 6 per group for both WT and GM^−/−^ mice (3 each gender); and 8 weeks post-MMS, n = 7 WT female mice and n = 5 GM^−/−^ female mice.

The μCT images of the middle coronal plane of the knee joints were reconstructed, and the long axis of the tibia was aligned using Amira software (Figure 3A). The correction angles along the long axes of the tibia for each bone are listed in Supplemental Data S1. The tibial width in IIOC, measured at the level of the growth plate when both the medial and lateral femoral condyles were clearly visualized, was significantly increased in WT mice at both 4 and 8 weeks following MMS compared with normal control joints (Figure 3B). The tibial IIOC height, calculated by counting μCT slices between the most proximal appearance of articular surface and growth plate, was reduced in WT mice significantly at both 4 and 8 weeks post-MMS compared to their normal control joints (Figure 3C).

We also normalized the maximal height of tibial IIOC by its width to enable cross-experimental comparisons. The results showed that the height-to-width ratio of tibial IIOC was significantly reduced at both 4 and 8weeks post-MMS (0.25 ± 0.02 and 0.24 ± 0.02, respectively, *p* < 0.01) compared to their contralateral normal joints (0.304 ± 0.011), but it did not show a difference between the two time points after MMS (Figure 3D). ROC analysis showed an AUC of 1.0 for both 4 and 8 weeks post-MMS, with a 95% CI of 1.0–1.0 (*p* < 0.01, Figure 3E). The cutoff value of <0.282 yielded 100% sensitivity and 100% specificity for both time points (Figure 3E). Importantly, the Bland–Altman analysis exhibited strong inter-rater reproducibility for the tibial IIOC height-to-width ratio in diagnosing MMS-induced OA, with an ICC of 0.887 (95% CI, 0.748–0.952) (Figure 3F).

At baseline (without MMS), both tibial width and maximal IIOC height were greater in GM^−/−^ mice than in WT mice (Figure 3B,C), whereas the tibial IIOC height-to-width ratio in normal joints did not differ between GM^−/−^ and WT mice (Figure 3D). Following MMS, tibial IIOC width remained unchanged at both 4 and 8 weeks (Figure 3B), and maximal IIOC height was unchanged at 4 weeks and showed only a marginal reduction at 8 weeks (Figure 3C), compared to the respective normal joint values. As a result, the tibial IIOC height-to-width ratio in GM^−/−^ mice did not differ at either 4 or 8weeks post-MMS compared with their normal joints (Figure 3D). Importantly, this ratio was significantly higher in GM^−/−^ mice than in WT mice at the corresponding 4- and 8-week time points following MMS (Figure 3D).

### 3.4. Geometric Parameters of Distal Femur and Proximal Tibia on μCT Images in Aged Mice

**Figure 4:**
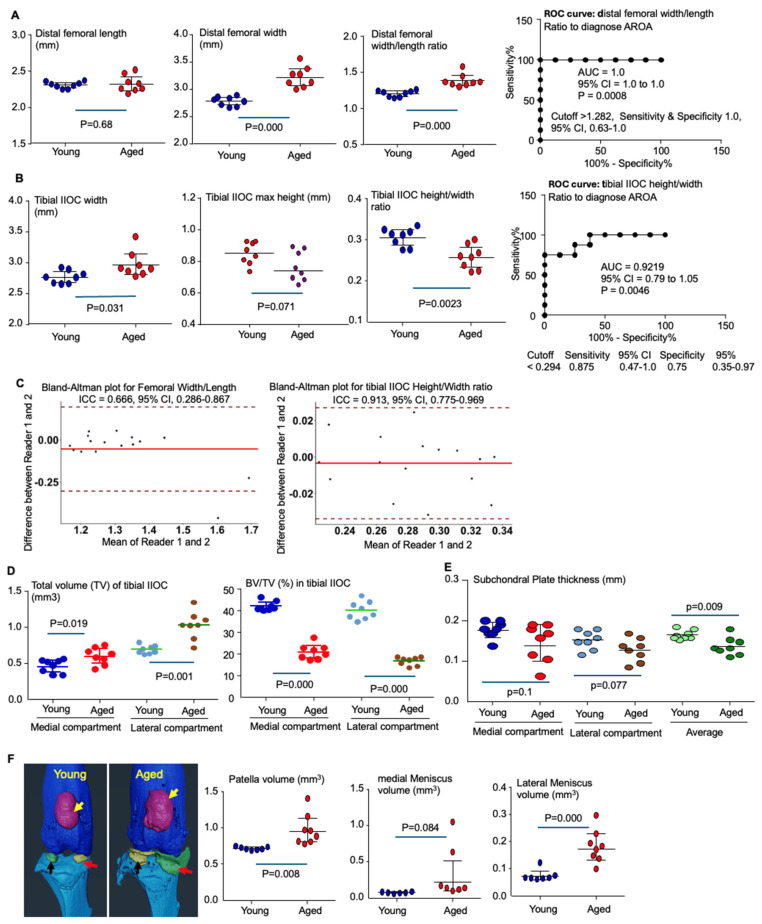
Alterations in geometric indices of distal femur and proximal tibia on μCT images of knee joints in aged mice. (**A**) Distal femoral length, width, and width-to-length ratio, measured on μCT images as illustrated in Figure 2B,E,F. ROC analysis showing the AUC and cutoff value of distal femoral width-to-length ratio in diagnosing AROA. (**B**) Tibial width, maximal height of tibial IIOC, and tibial IIOC height-to-width ratio, calculated as described in Figure 3B–D. ROC analysis showing the AUC and cutoff value of tibial IIOC height-to-width ratio in diagnosing AROA. (**C**) Bland–Altman plot and ICC for distal femoral width-to-length ratio and tibial IIOC height-to-width ratio in young and aged mice. (**D**) Total volume and BV/TV of tibial IIOC, quantified as in Figure 1C. (**E**) Subchondral plate thickness in the medial and lateral compartments of the tibial IIOC, measured as shown in Figure 1E. (**F**) Volume of patella (yellow arrows) and calcified synovium–capsule adjacent to medial meniscus (red arrows) and lateral meniscus (black arrows) in the knee joints of aged mice. Statistical analysis was performed using one-way ANOVA with Dunnett’s post hoc test. Sample number: 8 joints from 4 male mice for each young and aged group.

To evaluate the diagnostic utility of μCT-derived geometric indices from the distal femur and proximal tibia for AROA, we compared these indices in 28-month-old versus 5-month-old male C57Bl6 mice. The results indicated that 28-month-old aged mice exhibited a significant increase in distal femoral width but not in longitudinal length, resulting in a markedly higher ratio of distal femoral width to length compared with 5-month-old adult mice (1.39 ± 0.09 vs. 1.205 ± 0.045, *p* = 0.0004, Figure 4A). This ratio demonstrated strong diagnostic performance for AROA, with an AUC of 1.0 (95% CI, 1.0–1.0; *p* < 0.001; Figure 4A). A cutoff value of >1.282 yielded 100% sensitivity and 100% specificity (95% CI, 0.63–1.0). Consistent with these findings, the distal femoral width-to-length ratio remained below 1.27 in all young adult mice, whereas all joints from aged mice exceeded 1.3.

The aged mice also showed a significant increase in tibial IIOC width at the growth plate level, accompanied by a marginal reduction in the maximal height of the tibial IIOC, leading to a significantly decreased ratio of maximal IIOC height-to-tibial width compared with 5-month-old adult mice (0.307 ± 0.022 vs. 0.258 ± 0.029, *p* = 0.0023, Figure 4B). The tibial IIOC height-to-width ratio also exhibited strong diagnostic performance for OA, with an AUC of 0.9219 (95% CI, 0.79–1.05; *p* = 0.0046; Figure 4B). A cutoff value of <0.294 yielded 87.5% sensitivity (95% CI, 0.47–1.0) and 75% specificity (95% CI, 0.35–0.97).

Importantly, both the distal femoral width-to-length ratio and the tibial IIOC height-to-width ratio demonstrated strong interrater reproducibility for diagnosing AROA, with intraclass correlation coefficients of 0.667 (95% CI, 0.286–0.867) for the femoral ratio and 0.913 (95% CI, 0.775–0.969) for the tibial ratio (Figure 4C).

A multiplicity analysis indicated that both the distal femoral width-to-length ratio and the tibial IIOC height-to-width ratio remained consistent in normal joints from young adult mice (5 months old, aged controls) to middle-aged adult mice (10–12 months old, MMS controls) (Table 1 and Table 2). The effect size for the distal femoral width-to-length ratio was 0.75 (95% CI, 0.60–1.00), while the effect size for the tibial IIOC height-to-width ratio was 0.65 (95% CI 0.45–1.00). Notably, these ratios appeared to be stable across sex, as all eight joints from 5-month-old mice were from males, whereas the nine joints from 10–12-month-old mice included six females and three males. Consequently, the adjusted *p* values for both femoral and tibial ratios at 4 and 8 weeks post-MMS, when compared with normal joints from 5-month-old mice, were similar to those obtained when compared with their age-matched normal controls (Table 1 and Table 2). Similarly, the adjusted *p* values for both the femoral and tibial ratios in joints from aged mice were comparable when analyzed against either set of normal joints (Table 1 and Table 2). In addition, the mean values of both the femoral and tibial ratios in joints from aged mice were close to those observed in OA joints at 8 weeks post-MMS, although no statistically significant differences were detected when compared with either post-MMS time point.

The volumes of both the medial and lateral subchondral compartments in the IIOC of the tibia were significantly increased in 28-month-old aged mice compared with 5-month-old young adult mice (Figure 4D). In contrast, the BV/TV in both compartments was significantly reduced in aged mice relative to adult mice (Figure 4D), indicating the presence of osteoporosis in the aged group. Additionally, the thickness of the tibial subchondral bone plate was unchanged in the medial compartment but was marginally reduced in the lateral compartment of aged mice compared with young adult mice (Figure 4E).

We observed other common structural alterations in the knee joints of aged mice, including the ossification of peri-patellar tissues and the synovium adjacent to both the medial and lateral menisci, resulting in enlarged volumes of the patella and both menisci (Figure 4F).

### 3.5. Validation of the OA Geometric Parameters on μCT Images by Histological Analysis

**Figure 5:**
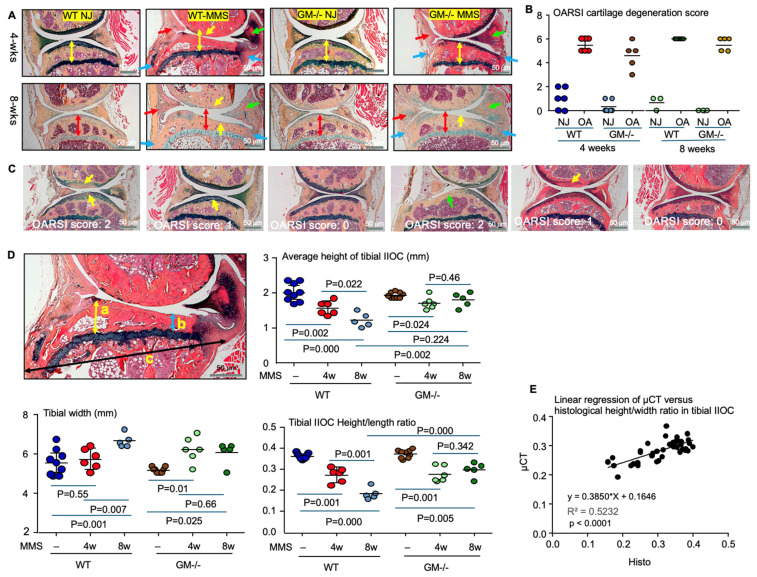
Histological evaluation of OA following MMS in WT and GM^−/−^ mice. (**A**) Representative images of Alcian Blue/H&E (ABH)-stained normal joints and OA joints at 4 and 8 weeks post-MMS. Red arrows: regeneration of the resected medial meniscus. Green arrows: enlargement of the contralateral meniscus due to endochondral ossification in the joint capsule. Blue arrows: osteophyte formation at the junction of synovium and capsule attached to the tibia. Yellow arrows: articular cartilage loss. Double-headed red arrows show the collapsed subchondral bone after MMS. (**B**) OARSI cartilage degeneration scores in normal and OA joints. (**C**) In 4 of 6 normal joints, “cartilage degeneration” was attributed to staining artifacts (yellow arrows) and slice-orientation issues (green arrow). (**D**) Histological measurements of the average height of tibial IIOC (mean of maximal height a, minimal height (b), and tibial length (c) at growth plate level, and tibial IIOC height-to-length ratio at 4 and 8 weeks after MMS. (**E**) Linear regression analysis of tibial IIOC height-to-width ratio derived from μCT versus histological measurement. Statistical analysis was performed using one-way ANOVA with Dunnett’s post hoc test. Sample sizes: 6 joints each for WT and GM^−/−^ mice at 4 weeks; 6 MMS joints for WT mice (one joint without exposure of the meniscus and cartilage was discarded due to orientation), 5 MMS joints for GM^−/−^ mice, and 3 normal joints for each genotype at 8 weeks.

Sagittal plane sections of the knee joints were subjected to Alcian blue/H&E (ABH) staining (Figure 5A) to blindly evaluate articular cartilage degeneration using the OARSI scoring system [33] by an experienced investigator. After 4 weeks of MMS, the articular cartilage degeneration scores were 5–6 in all knee joints of WT mice, whereas the scores ranged from 3 to 6 in GM^−/−^ mice, with no statistically significant difference between the two groups (Figure 5B). After 8 weeks of MMS, the cartilage degeneration score reached the maximum value of six in WT mice, while two out of five GM^−/−^ mice exhibited scores of four or five. One challenge in applying the OARSI scoring system is that four of six are normal. Uninjured knee joints were blindly assessed by an experienced investigator as having mild articular cartilage degeneration (score one or two) in WT mice (Figure 5C). This outcome may reflect technical factors such as sectioning quality, staining variability, or slice orientation.

We used histological images to validate the geometric parameters identified by μCT imaging that reflect osteophyte formation and subchondral bone plate collapse (cartilage degeneration). New bone formation at the junction between the synovium and the tibial bone was observed on both sides of the knee joint after 4 and 8 weeks of MMS (Figure 5A), resulting in an increased tibial width at the level of the growth plate (Figure 5C). Endochondral ossification was also observed at the sites of meniscal resection at both time points, leading to partial meniscal regeneration (Figure 5A). Additionally, endochondral ossification within the synovium and joint capsule after MMS contributed to an increased volume of the lateral meniscus in both WT and GM^−/−^ mice at 4 and 8 weeks (Figure 5A).

Histological analysis in WT mice at 4 weeks post-MMS revealed a marked decrease in the average height of the tibial IIOC, while the tibial width at the growth plate level was not changed (Figure 5D). This resulted in a significant reduction of the tibial IIOC height-to-width ratio compared to normal control joints (Figure 5D). By 8 weeks post-MMS, the tibial IIOC height continued to decrease, and the tibial width at the growth plate level had increased, together leading to a further, more pronounced decrease in the height-to-width ratio in WT mice (Figure 5D).

In GM^−/−^ mice, the average height of the tibial IIOC was decreased while the tibial width at the growth plate level was increased, resulting in a significant reduction of height-to-width ratio by 4 weeks post-MMS compared to the contralateral normal joints (Figure 5D). By 8 weeks post-MMS, both tibial height and width at the IIOC were maintained at the same value as those at 4 weeks post-MMS, thus preventing further worsening of the decreased tibial IIOC height-to-width ratio in GM^−/−^ mice (Figure 5D). Consequently, at this 8-week time point, the tibial IIOC height-to width ratio was higher in the knee joints of GM^−/−^ mice than that in WT mice (Figure 5D), consistent with observations from μCT images (Figure 3D). Importantly, the tibial IIOC height-to-width ratio measured histologically exhibited a strong linear correlation with those derived from μCT images in the normal and MMS-induced OA joints (Figure 5E).

**Figure 6:**
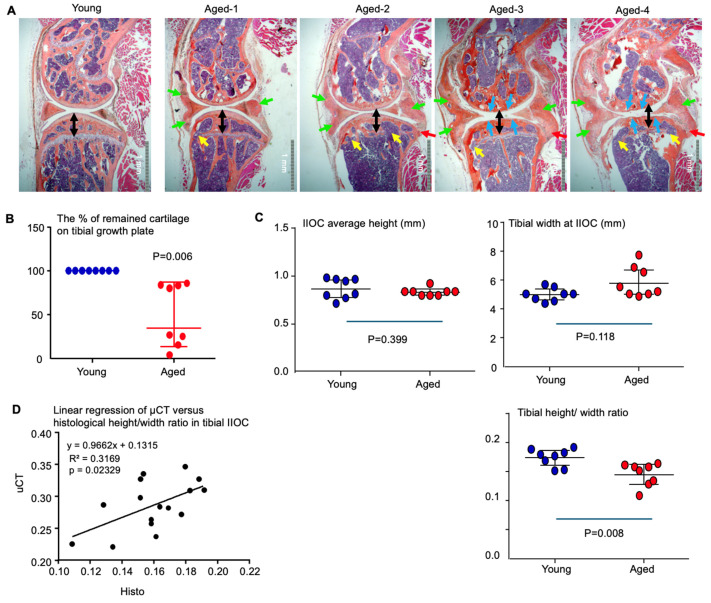
Histological evaluation of age-related OA. (**A**) Representative H&E-stained images of knee joints from young adult (5-month-old) and aged (28-month-old) mice. Double-headed black arrows indicate tibial IIOC height and areas of collapsed subchondral bone observed in some aged mice. Green arrows highlight ossification of the synovium and joint capsule adjacent to the meniscus. Red arrows indicate osteophyte formation at the junction of the synovium and capsule attached to the tibia. Blue arrows mark articular cartilage loss. Yellow arrows indicate loss of growth plate cartilage. (**B**) Assessment of growth plate cartilage loss in aged mice. (**C**) Histological measurements of average tibial IIOC height, width at growth plate level, and height-to-length ratio in young and aged mice, calculated as described in Figure 5D. (**D**) Linear regression analysis of tibial IIOC height-to-width ratio derived from μCT versus histological measurement. Statistical analysis was performed using ANOVA with Dunnett’s post hoc test. Sample size: 8 joints from 4 male mice for each young and aged group.

Lastly, we validated the geometric indices derived from μCT by histology for AROA. All 28-month-old mice consistently exhibited synovial and joint capsule ossification (Figure 6A), which aligned with μCT-based quantification of enlarged menisci (Figure 4E). However, the severity of articular cartilage degeneration, reduction of tibial IIOC height (subchondral plate collapse), and osteophyte formation in the knee joints were highly variable among these aged mice. In addition, they exhibited varying degrees of growth plate cartilage loss (Figure 6B). Based on these features, the disease severity can be categorized into the following types: (1) Normal (or Mild): normal articular cartilage and normal tibial IIOC height, slight synovial ossification without osteophyte formation at the synovium–tibia junction, and mild growth plate cartilage loss; (2) Moderate: mildly degenerated articular cartilage and collapsed tibial IIOC, moderate-to-severe synovial ossification with osteophyte formation at the synovium–tibia junction, and severe growth plate cartilage loss; and (3) Severe: severe articular cartilage degeneration and collapsed tibial IIOC, severe synovial ossification and osteophyte formation at the synovium–tibia junction, and mild-to-severe growth plate cartilage loss.

The heterogeneity of joint disease severity in aged mice likely explains why the tibial IIOC height and tibial length in 28-month-old mice did not differ significantly from those of young adult mice when assessed by histology (Figure 6C). In contrast, the tibial IIOC height-to-width ratio, quantified from histological sections, was significantly reduced in aged mice compared with young adults (Figure 6C), consistent with measurements obtained from μCT images (Figure 4B). This was further supported by a positive correlation between the histology-based and μCT-based height-to-width ratios (Figure 6D).

## 4. Discussion

This study is the first to apply the μCT-derived geometric indices of the distal femur and proximal tibia to assess the disease severity in both PTOA and AROP of murine knee joints. These indices were proven to be reproducible, sensitive, specific, and easily quantifiable. Increases in distal femoral width and in the distal femoral width-to-length ratio reflect osteophyte formation following joint injury and during age-related changes. This approach overcomes challenges in directly quantifying osteophytes, including the difficulty in defining boundaries between osteophytes and normal bone and the misidentification of periarticular sesamoid bones as separate osteophytes. Notably, the distal femoral width-to-length ratio enables robust comparisons across experiments. This ratio demonstrated 100% sensitivity and specificity for diagnosing MMS-induced OA (Figure 2G) and AROA (Figure 4A). Based on an ROC analysis, we propose a cutoff value of 1.28 for both PTOA and AROA, although this ratio in most normal joints from young and middle adult mice is less than 1.25, while it is greater than 1.29 in all PTOA and AROA joints. Knee joints can be considered normal when the distal femoral width-to-length ratio is less than 1.28, whereas a ratio greater than 1.28 indicates osteoarthritic changes driven by increased femoral width due to osteophyte formation in both PTOA and AROA (Figure 2F,G and Figure 4A). One challenging issue in measuring distal femoral length is to determine the midpoint of the upper femoral groove line in a few causes of OA, as the patella volume is increased, resulting in increased femoral groove too. Adjusting the threshold of Hounsfield Units may help to clearly identify the original boundary of the upper femoral groove.

The most commonly reported μCT-based parameters for assessing OA are structural indices within the tibial subchondral bone, such as BV/TV, trabecular number, and thickness [18,19,20]. These parameters reflect the often contradictory changes observed in subchondral bone, ranging from osteosclerosis in late-stage OA [18,19,20] to more common bone loss in early-stage OA [21,22]. In particular, the tibial subchondral bone in aged mice exhibited severe osteoporosis (Figure 4D), which does not reflect the major phenotype of OA. Changes in the μCT-derived geometric indices of the proximal tibia following OA in the knee joint reflect the complex pathogenesis involving cartilage degeneration, subchondral bone collapse, and osteophyte formation, which are validated by histological examination. The μCT image is more sensitive than histology to detect tibial osteophyte formation, showing significantly increased tibial width at 4 weeks post-MMS compared with the normal joints (Figure 3B), while it remained unchanged on the histology at this time point, probably due to the orientation of slices, as an outgrowth of new bone formation can be seen (Figure 5D). The μCT images can be rotated freely to identify the top edge of the bone on both sides. Similarly, the μCT image detects the elongation of the tibial width in the 28-months aged mice compared with the young adult mice (Figure 4B), due to the osteophyte formation at the junction between the synovium and the tibial bone (Figure 6A).

A more sensitive geometric parameter reflecting the pathological anatomy of the proximal tibia is the height of the IIOC, which is reduced at 4 and 8 weeks post-MMS, as detected by both μCT (Figure 3C) and histology (Figure 5D). A plausible explanation for the reduction in IIOC height is the collapse of the subchondral bone plate, resulting from the combined effects of cartilage degeneration and subchondral bone resorption [34], as supported by the following observations. First, the total volume of the tibial IIOC decreased significantly at 8 weeks post-MMS (Figure 1C) due to reduced volume of the medial subchondral compartment (Figure 1D). Second, the BV/TV in the tibial subchondral bone remained unchanged in general following MMS (Figure 1C), although the BV/TV in the medial subchondral compartment of the tibia decreased at 4 weeks post-MMS (Figure 1D). Third, the subchondral plate thickness of the tibia in both the medial and lateral compartments showed no significant difference between the MMS and control joints after 4 or 8 weeks (Figure 1E). The dense subchondral bone and thickened subchondral plate observed in the μCT and histologic images are likely due to slice orientation and the collapse of the subchondral plate, bringing it closer to the growth plate. This interpretation is supported by the reports that osteoclast activity is increased, with active bone resorption in the subchondral bone after MMS [21,34].

The height of tibial IIOC in aged mice not only reflects the pathogenesis of OA but also skeletal development, aging, and osteoporosis. Articular cartilage degeneration and the elongation of tibial width are the features of OA. Thinning and partial-to-complete loss of growth plate cartilage, occurring in 100% of 28-month-old mice, reflects growth plate closure through endochondral ossification, similar to the process observed in humans during adolescence. Loss of growth plate cartilage, along with resorption of adjacent cancellous bone, may lead to an apparent increase in the height of tibial IIOC, compensating for subchondral collapse and potentially resulting in inaccurate height measurements. The significantly lower BV/TV in the tibial IIOC (Figure 4D) and the thinner subchondral plate in aged mice compared to young adults (Figure 4E) indicate the presence of severe osteoporosis.

Similar to the distal femoral width-to-length ratio, we recommend using the tibial IIOC height-to-width ratio for inter- and across-experimental comparisons. This approach mitigates variability in absolute height or width that may arise due to individual and sex differences. This ratio demonstrated strong diagnostic performance for MMS-induced OA and AROA, with AUCs of 1.0 and 0.9219, respectively. For MMS-induced OA, we propose a tibial IIOC height-to-width ratio cutoff value of <0.276, yielding 92.3% sensitivity and 100% specificity. For AROA, a cutoff value of <0.282 is suggested, providing 87.5% sensitivity and 75% specificity. Importantly, the tibial IIOC height-to-width ratio showed strong inter-rater reproducibility for both MMS-induced OA and AROA.

It is important to note that not all joints in aged mice, or humans, exhibit an OA phenotype. Indeed, among our aged mice, three of eight knee joints with a tibial IIOC height-to-width ratio between 0.28 and 0.3 did not display major pathological features of OA on histological images, such as articular cartilage degeneration, subchondral plate collapse, or osteophyte formation at the synovium–bone junction. This suggests that, when the tibial IIOC height-to-width ratio falls within the range of 0.28 to 0.3, other indices should be considered for diagnosing AROA.

In addition to the structural modification in the distal femur and proximal tibia in AROA, the ossification of peri-patellar tissues and the synovium adjacent to both medial and lateral menisci, resulting in enlarged volumes of the patella and both menisci in the knee joints of 28 months aged mice (Figure 4E). This reflects that osteophytes are formed at the margins of the affected joints through endochondral ossification from the skeletal progenitors in the periosteum and synovium [35]. Similarly, an enlarged patella and regeneration of the resected meniscus and an enlarged, un-injured meniscus also occur in the knee joints after MMS (Figure 2D and Figure 5A). This is the first time showing that an enlarged patella and meniscus and a calcified synovium–capsule can serve as a reference for both AROA and PTOA, although it is known that osteophyte can form on the top of the patella [36], and ectopic ossification occurs in the synovium–capsule with severe OA joints [37].

Therapeutic responsiveness, the sensitivity to detect clinically meaningful changes in disease status after a treatment, is essential for a diagnosing tool. Given the absence of a cure for OA, we employed GM^−/−^ mice as a proof-of-concept model to test if μCT-based geometric indices can reflect therapeutic response, as GM^−/−^ mice have been reported to exhibit attenuated joint destruction and pain in collagenase-induced OA models [38,39,40,41]. Indeed, at 8 weeks post-MMS, increases in distal femoral width and tibial IIOC width were less pronounced in GM^−/−^ mice compared with WT mice, when each was compared to its respective contralateral normal joint. Consequently, the changes of both distal femoral width-to-length ratio and tibial IIOC height-to-width ratio were significantly mitigated compared with those in WT mice at 8 weeks post-MMS (Figure 2F and Figure 3D). These results suggest that both ratios are good parameters to assess the therapeutic response of OA treatment. Reduction of the distal femoral width-to-length ratio indicates less osteophyte formation. In contrast, an increase in the ratio of height to width of tibial IIOC suggests reduced articular cartilage degeneration, subchondral bone resorption and collapse, and osteophyte formation following PTOA.

In summary, μCT-based geometric indices, specifically the distal femoral width-to-length ratio and the tibial IIOC height-to-width ratio, are reliable, reproducible, and sensitive metrics for diagnosing OA both cross-sectionally and longitudinally, as well as for assessing disease severity and therapeutic responsiveness. An increased distal femoral width-to-length ratio reflects osteophyte formation resulting from enlargement of the distal femoral width. Conversely, a decreased tibial IIOC height-to-width ratio indicates articular cartilage degeneration, collapse of the subchondral plate driven by increased subchondral bone resorption, and osteophyte formation that expands tibial width. These geometric indices overcome challenges in directly quantifying osteophytes and reconcile contradictory μCT-based subchondral bone mass measurements, thereby improving the precision of OA diagnosis and disease severity assessment. In addition, they reduce the risk of misidentifying normal ossa sesamoidea near joints as osteophytes. Notably, both the distal femoral width-to-length ratio and the tibial IIOC height-to-width ratio remained stable from young to middle-aged adult mice and across sexes, suggesting that these geometric indices enable reliable comparisons across experiments.

It should be noted that the measurement of μCT-derived geometric indices depends on the identification of bony landmarks. To date, the distal femoral and proximal geometric indices have been validated only in knee joints with severe OA caused by MMS and by aging. These indices are expected to be applicable to other mild OA models, such as destabilization of the medial meniscus (DMM), provided that excessive bony outgrowth leading to enlargement of distal femoral and proximal tibial dimensions and/or subsequent subchondral bone collapse is present. Nonetheless, additional studies are required to validate their use in mild-to-moderately severe OA. A limitation of these geometric indices is that they do not directly quantify synovial inflammation or articular cartilage degeneration, although decreased tibial IIOC height reflects cartilage degeneration, subchondral bone resorption, and plate collapse. Further studies are needed to develop new indices that represent articular degeneration/erosion and synovial inflammation or ossification.

Several technical issues limit the measurement accuracy of μCT-based geometric indices of the distal femur and proximal tibia. First, measurements of very small anatomical structures in mice are limited by μCT resolution. For example, the subchondral plate thickness is only 100–150 μm, whereas the μCT voxel size is 10.5 μm, reducing measurement precision. Second, slice-number–based calculations become less accurate for tibial IIOC height when samples are scanned in a suboptimal orientation. Image orientations have been corrected to manually measure the tibial IIOC width. We attempted to measure tibial IIOC height manually using Amira software after correcting for tibial alignment along its long axis. However, rotations of the tibia along the other two axes substantially influenced these height measurements. Manual correction of alignment across all three axes proved to be time-consuming and unfeasible. Consequently, developing an AI tool for three-axial image orientation correction, based on clear anatomical landmarks, will be a focus of our future research. In aged mice, the growth plate is partially to completely absent, making precise measurement of tibial IIOC height challenging. Although the identification of additional anatomical landmarks may improve measurement accuracy, growth plate-based measurements remain applicable in aged mice because residual cartilage persists, and a sclerotic band can be identified at the site of the resorbed growth plate cartilage. The most challenging issue arises in adult humans, in whom the epiphyseal plate is closed, and the height of the tibial IIOC cannot be determined. Taken together, appropriate anatomical markers must be established to allow for consistent measurement of the distal femoral and proximal tibial dimensions in both animals and humans so that geometric indices can be accurately assessed and compared across investigators and study populations.

## 5. Conclusions

Distal femoral and proximal tibial geometric indices derived from μCT images demonstrate validity (authenticity), reproducibility (repeatability), and sensitivity (responsiveness to pathological or therapeutic changes) in diagnosing murine OA and assessing disease severity. The validity of these indices is supported by the observation that an increased distal femoral width-to-length ratio reflects enlargement of the distal femoral cross-sectional dimension resulting from osteophyte formation. In contrast, a decreased tibial IIOC height-to-width ratio reflects articular cartilage degeneration, subchondral bone collapse, and osteophyte formation. The inter-rater reproducibility of both ratios for evaluating PTOA and AROA has been confirmed. Proof of concept for the responsiveness of these ratios to therapeutic intervention was demonstrated in GM^−/−^ mice, given that there is currently no approved disease-modifying drug for OA. Based on our findings, the proposed cutoff values for diagnosing knee joint OA are a distal femoral width-to-length ratio greater than 1.29 and/or a tibial IIOC height-to-width ratio less than 0.29.

It should be noted that not all knee joints in aged mice or humans develop an OA phenotype. Therefore, OA diagnosis in joints with marginal geometric index values should also consider additional parameters, such as calcified meniscus and/or synovium–capsule and patellar enlargement, particularly in aged mice. To date, we have evaluated the utility of distal femoral and proximal tibial geometric indices only in MMS-induced OA and AROA models. We believe that these geometric indices may also be applicable to other OA models, as well as human OA.

## Figures and Tables

**Table 1:** Multiple group comparisons for distal femoral width-to-length ratio.

Group	Mean	SE	df	Lower.CL	Upper CL	*t* Ratio	*p* Value
WT Normal	1.19	0.0202	33	1.15	1.23	59.112	<0.0001
WT MMS 4 wk	1.33	0.0247	33	1.28	1.38	53.888	<0.0001
WT MMS 8 wk	1.42	0.0229	33	1.38	1.47	62.324	<0.0001
Young 5 month	1.21	0.0214	33	1.16	1.25	56.352	<0.0001
Aged 28 month	1.39	0.0214	33	1.34	1.43	64.874	<0.0001
**Contrast**			**estimate**	**SE**	**df**	***t* ratio**	***p* value**
(WT Normal)-(WT MMS 4 wk)			−0.1389	0.0319	33	−4.356	0.0002
(WT Normal)-(WT MMS 8 wk)			−0.2330	0.0305	33	−7.644	<0.0001
WT Normal)-(Young 5 month)			−0.0133	0.0294	33	−0.452	0.6543
(Aged 28 month)-(WT MMS 4 wk)			0.0567	0.0327	33	1.735	0.1152
(Aged 28 month)-(WT MMS 8 wk)			−0.0375	0.0313	33	−1.198	0.2662
(Aged 28 month)-(Young 5 month)			0.1822	0.0302	33	6.026	<0.0001
(WT MMS 4 wk)-(WT MMS 8 wk)			−0.0942	0.0337	33	−2.798	0.0122
(WT MMS 4 wk)-(Young 5 month)			0.1256	0.0327	33	3.844	0.0009
(WT MMS 8 wk)-(Young 5 month)			0.2197	0.0313	33	7.019	<0.0001
(WT Normal)-(Aged 28 month)			−0.1955	0.0294	33	−6.652	<0.0001

*p* value adjustment: BH method for 10 tests. SE = standard error; df = degrees of freedom; CL = confidence limit. Confidence level used: 0.95.

**Table 2:** Multiple group comparisons for tibial IIOC height-to-width ratio.

Group	Mean	SE	df	Lower CL	Upper CL	*t* Ratio	*p* Value
WT-Normal	0.304	0.00734	33	0.290	0.319	41.464	<0.0001
WT-MMS 4 wk	0.253	0.00899	33	0.235	0.271	28.153	<0.0001
WT-MMS 8 wk	0.241	0.00833	33	0.224	0.258	28.911	<0.0001
Young-5 month	0.307	0.00779	33	0.291	0.323	39.410	<0.0001
Aged-28 month	0.258	0.00779	33	0.242	0.274	33.152	<0.0001
**Contrast**			**estimate**	**SE**	**df**	***t* ratio**	***p* value**
(WT Normal)-(WT MMS 4 wk)			0.05128	0.0116	33	4.417	0.0002
(WT Normal)-(WT MMS 8 wk)			0.06375	0.0111	33	5.743	<0.0001
(WT Normal)-(Young 5 month)			−0.00247	0.0107	33	−0.231	0.8189
(Aged 28 month)-(WT MMS 4 wk)			0.00502	0.0119	33	0.422	0.7511
(Aged 28 month)-(WT MMS 8 wk)			0.01749	0.0114	33	1.534	0.1923
(Aged 28 month)-(Young 5 month)			−0.04874	0.0110	33	−4.425	0.0002
(WT MMS 4 wk)-(WT MMS 8 wk)			0.01247	0.0123	33	1.017	0.3954
(WT MMS 4 wk)-(Young 5 month)			−0.05375	0.0119	33	−4.518	0.0002
(WT MMS 8 wk)-(Young 5 month)			−0.06622	0.0114	33	−5.809	<0.0001
(WT Normal)-(Aged 28 month)			0.04627	0.0107	33	4.322	0.0002

*p* value adjustment: BH method for 10 tests. SE = standard error; df = degrees of freedom; CL = confidence limit. Confidence level used: 0.95.

## Data Availability

Raw data for the geometric indices are included in Supplemental Tables S1–S3.

## Supplementary Materials

The following supporting information can be downloaded at https://www.mdpi.com/article/10.3390/biology15030262/s1: Table S1: reported μCT data in proximal tibia (subchondral bone); Table S2: corrected angle of tibial orientation for manual measurement of proximal tibial indices; Table S3: geometric indices from distal femur and proximal tibia.
